# Minocycline improves the functional recovery after traumatic brain injury via inhibition of aquaporin-4

**DOI:** 10.7150/ijbs.64187

**Published:** 2022-01-01

**Authors:** Qi Lu, Jun Xiong, Yuan Yuan, Zhanwei Ruan, Yu Zhang, Bo Chai, Lei Li, Shufang Cai, Jian Xiao, Yanqing Wu, Peng Huang, Hongyu Zhang

**Affiliations:** 1 School of Pharmaceutical Sciences, Wenzhou Wound Repair and Regeneration Key Laboratory, Cixi Biomedical Research Institute, Wenzhou Medical University, 325000, Wenzhou, Zhejiang, China.; 2 Department of pharmacy, Hangzhou Red Cross Hospital, Zhejiang Province Hospital of Integrated Traditional Chinese and Western Medicine, 310003, Hangzhou, Zhejiang, China.; 3 Department of Emergency, Ruian People's Hospital, The Third Affiliated Hospital of Wenzhou Medical University, 325000, Wenzhou, Zhejiang, China.; 4 The Institute of Life Sciences, Engineering Laboratory of Zhejiang Province for Pharmaceutical Development of Growth Factors, Biomedical Collaborative Innovation Center of Wenzhou, Wenzhou University, 325035, Wenzhou, Zhejiang, China.; 5 Department of Pharmacy, Ruian People's Hospital, The Third Affiliated Hospital of Wenzhou Medical University, 325200, Wenzhou, Zhejiang, China.; 6 Department of Pharmacy, Zhuji People's Hospital, The Affiliated Hospital of Wenzhou Medical University, 311899, Shaoxing, Zhejiang, China.

**Keywords:** Traumatic brain injury (TBI), Minocycline, Aquaporin-4 (AQP4), Blood-brain barrier (BBB), Astrocytes

## Abstract

Traumatic brain injury (TBI) is one of the main concerns worldwide as there is still no comprehensive therapeutic intervention. Astrocytic water channel aquaporin-4 (AQP-4) system is closely related to the brain edema, water transport at blood-brain barrier (BBB) and astrocyte function in the central nervous system (CNS). Minocycline, a broad-spectrum semisynthetic tetracycline antibiotic, has shown anti-inflammation, anti-apoptotic, vascular protection and neuroprotective effects on TBI models. Here, we tried to further explore the underlying mechanism of minocycline treatment for TBI, especially the relationship of minocycline and AQP4 during TBI treatment. In present study, we observed that minocycline efficaciously reduces the elevation of AQP4 in TBI mice. Furthermore, minocycline significantly reduced neuronal apoptosis, ameliorated brain edema and BBB disruption after TBI. In addition, the expressions of tight junction protein and astrocyte morphology alteration were optimized by minocycline administration. Similar results were found after treating with TGN-020 (an inhibitor of AQP4) in TBI mice. Moreover, these effects were reversed by cyanamide (CYA) treatment, which notably upregulated AQP4 expression level *in vivo*. In primary cultured astrocytes, small-interfering RNA (siRNA) AQP4 treatment prevented glutamate-induced astrocyte swelling. To sum up, our study suggests that minocycline improves the functional recovery of TBI through reducing AQP4 level to optimize BBB integrity and astrocyte function, and highlights that the AQP4 may be an important therapeutic target during minocycline treating for TBI.

## Introduction

Traumatic brain injury (TBI), an organic cerebral injury from external forces, remains to be one of the main health challenges; it contributes to multiple neuropathological complications as well as long-term neurodegenerative processes among all ages 1, 2. TBI involves the intricate mechanisms and a cascade network that ranges from physical primary cerebral trauma to subsequent series of secondary injuries. The above pathological processes are considered to be of vital importance in functional recovery following TBI 3. Brain edema, Blood-Brain Barrier (BBB) disruption, vascular dysfunction and glial responses have been shown to bound up with the development of the secondary injuries following TBI, ultimately leading to irreversible neuronal injury or even death 4, 5. Notably, various types of cells, including neurons, endothelial cells and astrocytes, are implicated in the pathological processes of TBI. It has been reported that astrocytes distribute predominantly in the brain, which serves as crucial roles in the regulation of water homeostasis, maintenance of BBB integrity, and gliovascular coupling 6-8.

Aquaporin-4 (AQP-4), the most abundant water channel protein in the CNS, is primarily expressed in perivascular astrocyte end-feet processes at the BBB, and may function as a key modulator for astrocyte properties 9, 10. Astrocyte reactivation, migration and proliferation are found to be positively correlated with AQP4 gene expression, suggesting a fundamental mechanism in CNS disorders 11. Previous studies have demonstrated that the elevated expression of AQP4 after spinal cord compression injury results in spinal cord edema (tissue swelling) and excessive neuronal death, finally exacerbating neurological deficits, which of all these deficits are partly reversed in AQP4-null mice 12. As a complement of BBB properties, astroglial AQP4 has been shown to regulate barrier permeability via astrocyte-related tight junction formation and brain water transport 13, 14. Further evidences also suggest that astrocytic AQP4 distribution contributes to the gliovascular stability with the features of microvessel integrity and astrocyte morphological alterations in post-stroke dementia 15 as well as white matter damage after mild TBI 16.

As a clinically available tetracycline-antibiotic, minocycline has already exhibited a considerable neuroprotective effect on numerous models of neurological disorders 17. Its high lipophilicity and BBB penetration make it promising for intervening in the secondary injury via anti-inflammatory, anti-apoptotic, anti-oxidant and vasculature protective properties 18, 19. To date, most studies have been focused on the anti-inflammatory effect of minocycline on microglial activation in the CNS 20-22. However, the study targeted on AQP4 regulation of minocycline treatment for acute TBI is still lacking. It has been reported that minocycline preserves BBB integrity through altering the cell secretion-related signaling in intracerebral hemorrhage (ICH) rat model 23. Moreover, minocycline up-regulated the levels of tight junction proteins and promotes BBB remodeling after cerebral ischemia injury 24. Additional studies also suggested that minocycline elevates the expressions of angiogenesis promoters in vascular endothelial cells, contributing to cerebral vasculature remodeling in white matter repair 25. In this study, we found that in accordance with the previous investigation, minocycline treatment restored the AQP4 upregulation at 3 days after TBI 26. Thus, we propose that minocycline-related AQP4 reduction may underlie astrocyte function optimization and subsequent protection of BBB, unveiling the molecular mechanisms for minocycline treatment in TBI.

In the present study, we showed that minocycline has a neuroprotective role for acute TBI, as indicated by the remission of cell apoptosis and BBB disruption. Further research suggested that the expression and location of AQP4 is related to the neuroprotective role of minocycline. Based on the comparative analysis of the pharmacological effects of minocycline and TGN-020 (an AQP4 inhibitor), we identified that these two compounds regulated astrocyte properties and BBB function via similar mechanisms. Meanwhile, we demonstrated that CYA (an AQP4 agonist) administration partly reverses the effects of minocycline and TGN-020 in regulating astrocyte properties and BBB function. In sum, this study unraveled a specific role of AQP4 in minocycline treatment for TBI.

## Materials and methods

### Animals and drug administration

Male wild-type C57BL/6J mice (6-8weeks old; 20-25 g body weight) were purchased from GemPharmatech Co., Ltd (Nanjing, China), and were housed under controlled environmental condition (12 h light/dark cycles, 24±1°C) with ad libitum access to food and water. Mice were divided into six groups: Sham group, TBI group, TBI+Mino group, TBI+Mino+CYA group, TBI+CYA group and TBI+TGN-020 group. All procedures were performed in accordance with the guidelines from Laboratory Animal Care and Use Committee and Animal Ethics Committee of Wenzhou Medical University. For drug administration, minocycline hydrochloride (Sigma-Aldrich, St. Louis, MO, USA) was dissolved in saline (9 mg/mL) and intraperitoneal (i.p.) injected at the dose of 45 mg/kg 30 min after TBI. CYA (Sangon Biotech, Shanghai, China) and TGN-020 (Sigma-Aldrich, St. Louis, MO, USA) were dissolved in normal saline solution and used for the regulation of AQP4. TGN-020 was i.p. injected at the dose of 200 mg/kg 30 min after TBI, while CYA was i.p. injected at the dose of 100 mg/kg 60 min after TBI (CYA needs delayed injection for its combination use with Mino). These three drugs were injected with same doses at 24 h intervals before mice sacrifice. The efficacy and safety of these doses in mice have been demonstrated previously 27, 28. All efforts were made to minimize the pain and prevent infections.

### Induction of traumatic brain injury model

The surgical procedures to set up TBI model by controlled cortical impact (CCI) device were performed as previously described 29. Briefly, male mice were anesthetized with 3% isoflurane (RWD Life Science, Shenzhen, China) and with maintenance in 1.5%. Mice were fixed in a sterile stereotaxic apparatus and a midline incision was made to expose the cranium. A 5 mm craniotomy was performed aseptically by a dental drill on the center of left parietal bone. After careful removal of bone flap, TBI was induced by Impact One™ stereotaxic impactor (Leica, Milan, Italy) using a 4-mm impact tip. The parameters of device were set as 4 m/s of impact velocity, 0.9 mm of impact depth and 150 ms of impact duration. After surgery, the incision was sutured and sterilized immediately. The mice in sham group were received all surgical procedures except cortical impact. Mice were then placed on a 37°C heating pad to maintain the body temperature during anesthetic period and conducted the drug administration as described above.

### Sodium fluorescein penetration assay

The fluorescent tracer sodium fluorescein (NaF, 376 Da, Sigma-Aldrich, MO, USA) was dissolved in saline (20 mg/mL) and i.p. injected at the dose of 100 mg/kg (2 mg/mice) to evaluate the BBB leakage stated previously 30. Compared with Evans blue dye (961 Da), its smaller molecular weight (376 Da) offered increased sensitivity in permeability detection 31. After 5 h circulation, the mice were sacrificed and whole brain was collected. Fluorescence intensity was measured by CRI Maestro™ spectrum imaging system (CRI Corporation, MA, USA). For quantitative assay, the left hemisphere cortex was dissected and incubated in trichloroacetic acid solution (50% w/v in purified water) at room temperature for 72 h. After centrifuge at 13600 g for 10 min, 200 μL supernatant of each samples were collected in 96-well plate. The fluorescence of NaF was measured at 485 nm excitation and 530 nm emission by microplate reader (Bio-Rad, CA, USA). Optical density (OD) was corrected for background and transformed into concentration using NaFI standard curve.

### Measurement of water content in brain

Mice were anesthetized and sacrificed to dissect the brain immediately. For a more precise cerebral edema measurement, the cerebellum and olfactory bulbs were removed and weighed to obtain the wet weight. Then, the brain samples were dried at 70°C for 72 h and weighed to obtain the dry weight. Brain water content (%) was calculated as [(wet weight- dry weight)/wet weight] × 100%.

### Fluoro-Jade B (FJ-B) staining

FJ-B staining (Millipore, CA, USA) is a sensitive fluorescein, which used to detect degenerating and apoptotic neuron 32. 5 μm brain paraffin-embedded sections were selected and removed paraffin by xylene, then rinsed twice with alcohol. Sections were typically coated with 2% gelatin and air dried for half an hour at 50°C. Then the slides were rinsed in 70% (v/v) alcohol for 2 min, distilled water for 1 min and transferred to 0.06% (w/v) potassium permanganate solution for 10 min on the shaker table. After rinsing 2 min in distilled water, the slides were immersed in 0.0002% (w/v) staining solution with 0.1% (v/v) acetic acid vehicle at room temperature for 20 min. The slides were rinsed for 1 min in each of three distilled water wash and fully dried at 50°C (5-10 min). The dry slides were again rinsed in xylene for 1 min before coverslipping with DPX (Sigma, St. Louis, MO, USA). FJ signals were then examined at 480 nm excitation and 525 nm emission by confocal microscope (Nikon, A1 PLUS, Tokyo, Japan).

### TUNEL assay

TUNEL Apoptosis Detection Kit (Yeason, Shanghai, China) was used to detect the DNA fragmentation and cell apoptosis. Briefly, the brain sections were deparaffinized, rehydrated and rinsed by PBS. Then, 100 μL of 20 μg/mL Proteinase K solution was added to each section and incubated for 20 min at room temperature. TdT incubation buffer solution was made up with 5×Equilibration Buffer, Alexa Fluor 488-12-dUTP Labeling Mix and Recombinant TdT Enzyme. The sections were incubated in the solution for 60 min at 37°C incubator. After rinsing with PBS, the sections were coverslipped with fluorescent mounting medium with DAPI (Yeason, Shanghai, China). The images were captured by the Nikon ECLIPSE Ni microscope (Nikon, A1 PLUS, Tokyo, Japan). TUNEL-positive cells in five random fields were quantified by Image J for analysis.

### Primary cortical astrocyte culture and drug treatment

Primary astrocytes were obtained from newborn Sprague-Dawley (SD) rats (P2-P3) as previously described 33. Briefly, the neonatal rats were sterilized in 75% alcohol and euthanized by decapitation. The cerebral cortex was separated and transferred into ice-cold Hank's Balanced Salt Solution (Gibco, Grand Island, USA). The meninx and capillaries were removed under the microscope before the cortices were fragmented and dissociated with pre-warmed 0.0125% trypsin (Gibco, USA) for 20 min. After 70 μm filter of filtration, the cell suspension was centrifuged at 1000 rpm for 5 min and supernatant was discarded. Cells were resuspend in DMEM/F12 (Gibco, USA) supplemented with 10% fetal bovine serum (FBS, Gibco, USA) and seeded on culture flasks pre-coated with poly-L-lysine (Sigma-Aldrich, St. Louis, MO, USA). Cells were maintained at 37°C humidified incubator with 5% CO_2_. Cell medium was changed after first 24 h and then every 2-3 days. After 7-14 days, cells were shaken at 200-240 rpm for at least 6h to remove microglia and oligodendrocytes. Cells were subcultured for three generations to obtain purified cultured astrocytes, which were examined by immunofluorescence of glial fibrillary acidic protein (GFAP, Abcam, Cambridge, MA). To evaluate the role of AQP4 in astrocytes, cells were pretreated with minocycline (20 μM) for 2 h, and then incubated with glutamate (1 mM) for 48 h. If cells needed siRNA transfection, the drug administration should be after it.

### Cell viability assay

Cell viability was detected by Cell Counting Kit-8 (CCK-8) assay kit. Primary cultured astrocytes were seeded on 96-well plates at a density of 8 × 10^3^ cells per well and incubated at 37°C overnight. After treated with minocycline and glutamate separately, 10 μL CCK-8 reagent was added into 100 μL culture medium per well and incubated at 37°C for 2 h. Optical density (OD) was measured at 450 nm.

### Small-interfering RNA knockdown of AQP4

3 different sequences of siRNA (AQP4-402, AQP4-653, AQP4-725) identical to rat AQP4 were designed and synthesized by Sangon Biotech, Shanghai, China. The AQP4 knockdown efficiency was tested and the optimum concentration is 50 nM. Primary cultured astrocytes were seeded on 12-well plates at the density of 2.5×10^4^ cells/well using antibiotic-free DMEM/F12 medium with 10% FBS and cultured for 24 h. Before transfection, cell culture medium was replaced by Opti-MEM (Gibco, USA). AQP4 siRNA and siRNA scramble were diluted by Opti-MEM (2.5 μL siRNA/100 μL Opti), and mixed up respectively with lipofectamine 2000 reagent (2 μL lip/100 μL Opti) (Thermo Fisher Scientific, MA, USA) incubating at room temperature for 20 min. The mixed solutions were added into plates and transfected for 6 h. The specific AQP4 knockdown was checked by western blot assay. The rat AQP4 siRNA sequences were listed as below: AQP4-402, sense 5'-CCAAGUCCGUCUUCUACAUTT-3', antisense 5'-AUGUAGAAGACGGACUUGGTT-3'; AQP4-653, sense 5'-CCGUUGCAAUUGG ACUUUTT-3', antisense 5'-AAAUGUCCAAUUGCAACGGTT-3'; AQP4-725, sense 5'-GCAG UUAUCAUGGGAAACUTT-3', antisense 5'-AGUUUCCCAUGAUAACUGCTT-3'.

### Western blot assay

Left cerebral cortex (lesion side) of brain tissue was dissected at day 3 after TBI, and homogenized in RIPA lysis buffer containing protease inhibitor cocktail. After centrifuge at 12000 g, 4°C for 15 min, the proteins from supernatant were quantified by bicinchoninic acid reagents (BCA, Thermo, Rockford, I L, USA). Equivalent proteins (60 μg) were separated on 10% or 12% SDS-PAGE gels for electrophoresis and transferred onto PVDF membrane (Bio-Rad, Hercules, CA, USA). Membranes were then blocked in 5% (w/v) skim milk in TBS with 0.1% Tween-20 (TBST) for 1.5 h at room temperature and incubated with following primary antibody at 4°C overnight: cleaved-caspase 3 (1:500, Affinity), Bax (1:1000, Abcam), Bcl-2 (1:1000, Cell Signaling Technology, MA, USA), Albumin (1:1000, Abcam), ZO-1 (1:1000, Abcam), Occludin (1:1000, Proteintech), Claudin-5 (1:1000, Invitrogen), MMP-9 (1:1000, Abcam), AQP4 (1:400, Sigma), GFAP (1:1000, Abcam) and GAPDH (1:5000, Multisciences). The membranes were rinsed 3 times with TBST and incubated with horseradish peroxidase-conjugated rabbit/mouse secondary antibodies (1:10000, Multisciences) for 60 min at room temperature. The band signals were visualized using ChemiDoc^TM^ XRS imaging system (Bio-Rad, United States). The relative band densities were analyzed by Image Lab 5.2 software (Bio-Rad). All experiments were repeated independently at least 3 times.

### Immunofluorescence and immunohistochemistry

The brain was fixed in 4% paraformaldehyde (PFA) at 4°C for 24 h-48 h, then dehydrated with gradient concentration of alcohol and embedded with paraffin. 5 μm brain sections were removed paraffin, rehydrated and rinsed by PBS. Sections were blocked by 5% bovine serum albumin (BSA) in 37°C for 30 min. Then sections were incubated with following primary antibodies (diluted with 1% BSA) in 4°C overnight: goat anti-CD31 (1:200, R&D system), rabbit anti-ZO-1 (1:200, Abcam), rabbit anti-albumin (1:500, Abcam), mouse anti-GFAP (1:1000, Abcam), rabbit anti-AQP4 (1:200, Sigma), rabbit anti-cleaved caspase 3 (1:250, Affinity). After 3 time rinsing with PBS, the sections were incubated with Alexa-Fluor 488, 547 and 647 donkey anti-mouse/rabbit secondary antibody (1:1000, Abcam) in 37°C incubator for 1 h. Finally, the slices were washed by PBST and coverslipped with fluorescent mounting medium with DAPI (Yeason, Shanghai, China). The images were captured by Nikon ECLIPSE Ti microscope (Nikon, Tokyo, Japan). To assess the astrocyte morphology *in vitro*, astrocytes were fixed by 4% PFA at room temperature for 20 min, rinsed 3 times by PBS, and incubated with 5% BSA containing 0.1% Triton X-100 for 30 min in 37°C incubator. The following steps were the same as above. For immunohistochemistry, the brain sections were deparaffinized, rehydrated, rinsed and blocked by 5% BSA for 30 min incubation. Sections were stained with hematoxylin after incubated with primary antibody (GFAP) and secondary antibody. The images were captured by Nikon ECLIPSE Ni microscope (Nikon, Tokyo, Japan) with bright field.

### Statistical analysis

Data were presented as mean ± SEM from at least 3 independent experiments. Statistical significance was analyzed using one-way analysis of variance (ANOVA) followed by Tukey's post hoc test for multiple comparisons. All analysis was conducted by GraphPad Prism 6 and statistical significance was defined as *P*< 0.05. Each experiment was performed at least 3 times to ensure accuracy, and the tissues from each replicate were from different mice.

## Results

### Minocycline reduces neuronal apoptosis after TBI

**Figure 1:**
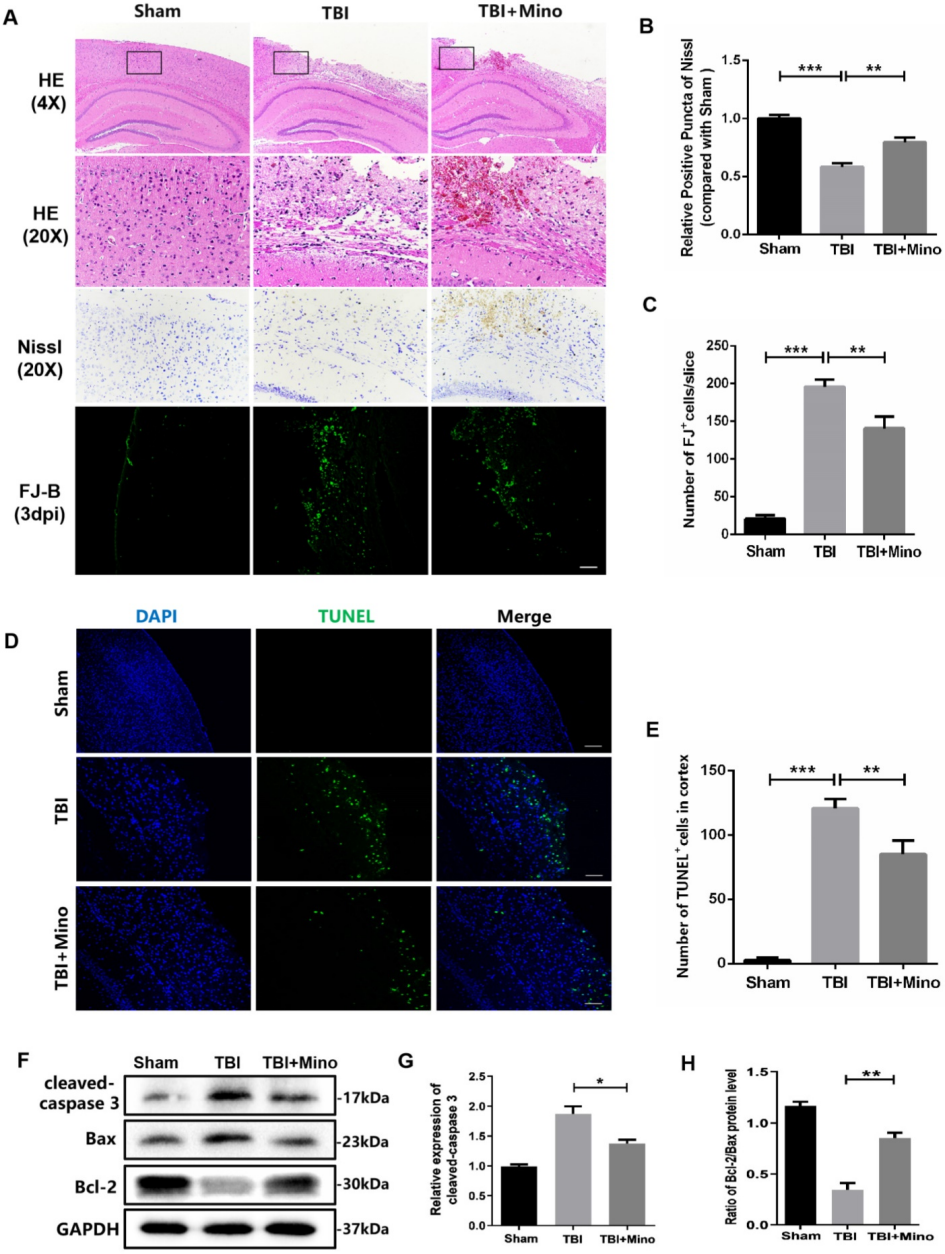
**Minocycline reduces neuronal loss and exerts neuroprotective role after TBI. (A)** The representative images of H&E, Nissl and FJ-B staining of ipsilateral cerebral cortex at day 3 from Sham, TBI and TBI+Mino group. Scale bar= 500 µm (4×), Scale bar=100μm (20×)**. (B)** Quantification of Nissl staining for neuronal loss analysis at 3 day after TBI. **(C)** Quantification of FJ-B immunofluorescence staining for neuronal apoptosis analysis at 3 day after TBI. **(D)**Representative images of TUNEL (green) immunofluorescence staining in perilesional cortex 3 day post-TBI. Scale bar= 100 µm.** (E)** Quantification of TUNEL staining at 3 day after TBI. **(F)** Representative western blot analysis of cleaved-caspase 3, Bax and Bcl-2 in ipsilateral cerebral cortex 3 day post-TBI. **(G, H)** Quantification of cleaved-caspase 3 and the ratio of Bcl-2/Bax from western blot analysis. All data represent the mean ± SEM, n = 3, *P < 0.05, **P < 0.01, ***P < 0.001 *vs.* the indicated group.

Firstly, we determined the therapeutic effect of minocycline on acute TBI. Hematoxylin & Eosin (H&E) staining was performed to evaluate the histological morphology of cerebral cortex. As shown in Figure 1A, minocycline administration significantly alleviated the TBI-induced cerebral cortex damage and loss, as well as improving the looseness of cortical tissue. Given that neuronal apoptosis plays a vital role in the secondary injury after TBI, we examined the neuronal loss in cerebral cortex by using TUNEL, Nissl and FJ-B staining. Notably, minocycline treatment led to the inhibition of cell apoptosis (TUNEL positive cells) in the cortex after TBI, as indicated by representative images and quantification of TUNEL staining (Figure 1D, E). Meanwhile, Nissl and FJ-B staining revealed that the significantly increased number of Nissl bodies and reduced dying neurons in perilesional cortex are present in mice from the TBI + minocycline group compared with that in the TBI group, showing that minocycline treatment markedly attenuates TBI-induced neuronal apoptosis (Figure 1A-C). Consistent with the staining assays described above, minocycline treatment following TBI down-regulated the expression of cleaved-caspase 3 and up-regulated the ratio of Bcl-2/Bax in the cortex from TBI group, as shown in the western blotting analysis (Figure 1F-H). Collectively, our data indicated that minocycline has a neuroprotective role during treatment for TBI mice.

### Minocycline attenuates BBB dysfunction following TBI

**Figure 2:**
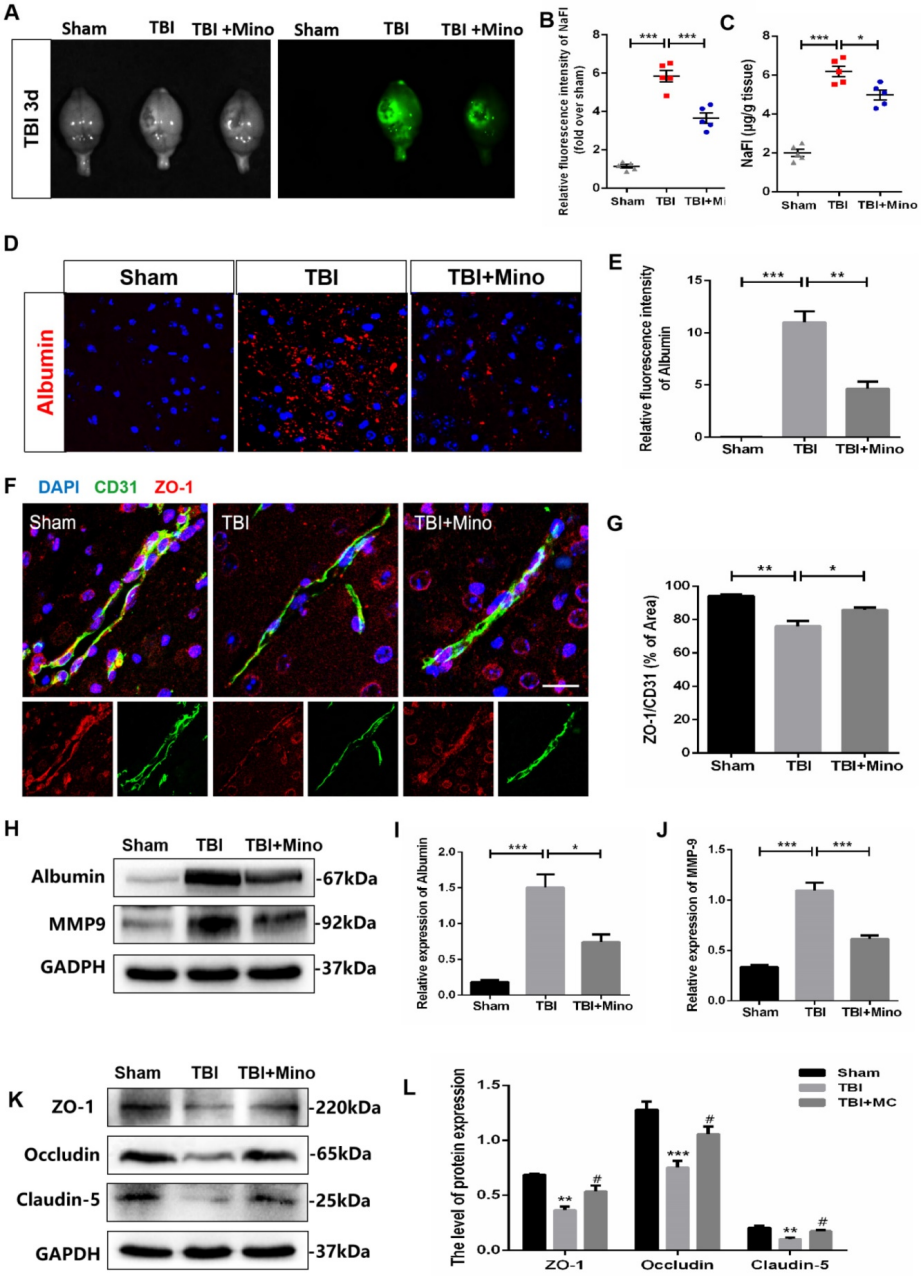
** Minocycline attenuates BBB dysfunction after TBI. (A)** Representative images of fluorescent tracer sodium fluorescein (i.p. injection) leakage to visualize BBB disruption 3 day after TBI. Images of bright field (left) and fluorescent field (right) come from the same brains of each group. **(B)** Quantification of the relative fluorescence intensity of sodium fluorescein (NaFI). NaFI standard curve: y=6309.6x-263.5, R^2^= 0.9999. **(C)** Quantification of sodium fluorescein leakage (µg) per gram of the brain tissue from mice. n=5 mice per group. *P < 0.05, ***P < 0.001 *vs.* the indicated group.** (D)** Immunofluorescence images of Albumin (red) in perilesional cortex to indicate BBB disruption at 3 dpi. Scale bar= 50 µm, n= 3 per group.** (E)** Quantification of relative fluorescence intensity of Albumin. **P < 0.01, ***P < 0.001 *vs.* the indicated group**. (F)** Representative immunofluorescence images depicting ZO-1 (red) with CD31 (green) at 3 dpi. Scale bar = 25 µm, n= 3 per group. **(G)** Co-localization analysis of the ZO-1^+^ area and CD31^+^ area. *P < 0.05, **P < 0.01 *vs.* the indicated group. **(H-J)** Representative western blot analysis and quantification of Albumin and MMP-9 in ipsilateral cortex 3 day after TBI. n = 3 per group. *P < 0.05, ***P < 0.001 *vs.* the indicated group.** (K-L)** Representative western blot analysis and quantification of tight junction proteins (ZO-1, Occludin and Claudin-5) 3 day after TBI. n = 3 per group. **P < 0.01, ***P < 0.001 *vs.* Sham group, ^#^P < 0.05 *vs.* the TBI group. All data represent the mean ± SEM.

To assess the role of minocycline treatment for BBB integrity following TBI, we analyzed the leakage of the fluorescent tracer sodium fluorescein (i.p injected) in the whole brain at 3 dpi. The bright field and fluorescent field of representative brain images were captured by CRI Maestro™ spectrum imaging system, and we determined their relative fluorescence intensities as well as the content of NaFI (μg/g brain tissue) (Figure 2A). As depicted in Figure 2B and 2C, minocycline significantly suppressed the enhancement of fluorescence intensity and reduced the leakage of sodium fluorescein in the whole brain following TBI. In the meantime, we found that minocycline treatment inhibits TBI-caused the enhancement of Albumin extravasation (Figure 2D, E). Likewise, western blot analysis revealed that minocycline administration following TBI markedly suppresses the expressions of Albumin and MMP-9 (Figure 2H-J). All these results demonstrated that minocycline alleviates BBB dysfunction following TBI. To further investigate the mechanism underlying TBI-caused BBB breakdown, we examined tight junctions in the ipsilateral cortex, which plays an essential role between the endothelial cells and astrocyte end feet 34. Compared with Sham group, TBI group displayed a loss of tight junction protein (ZO-1) and endothelial coverage, as indicated by the co-immunostaining of ZO-1 (tight junction marker) and CD31 (vascular endothelial marker). Notably, minocycline treatment following TBI significantly enhanced the ZO-1 expression and alleviated the vascular dysfunction (Figure 2F, G). Western blot analysis showed that minocycline treatment restores the expressions of tight junction proteins (ZO-1, Occludin and Claudin-5) following TBI (Figure 2K, L). Together, these findings suggested that minocycline attenuates BBB disruption partly through restoring the tight junction proteins after TBI.

### Minocycline inhibits AQP4 expression and lessens the cerebral edema after TBI

**Figure 3:**
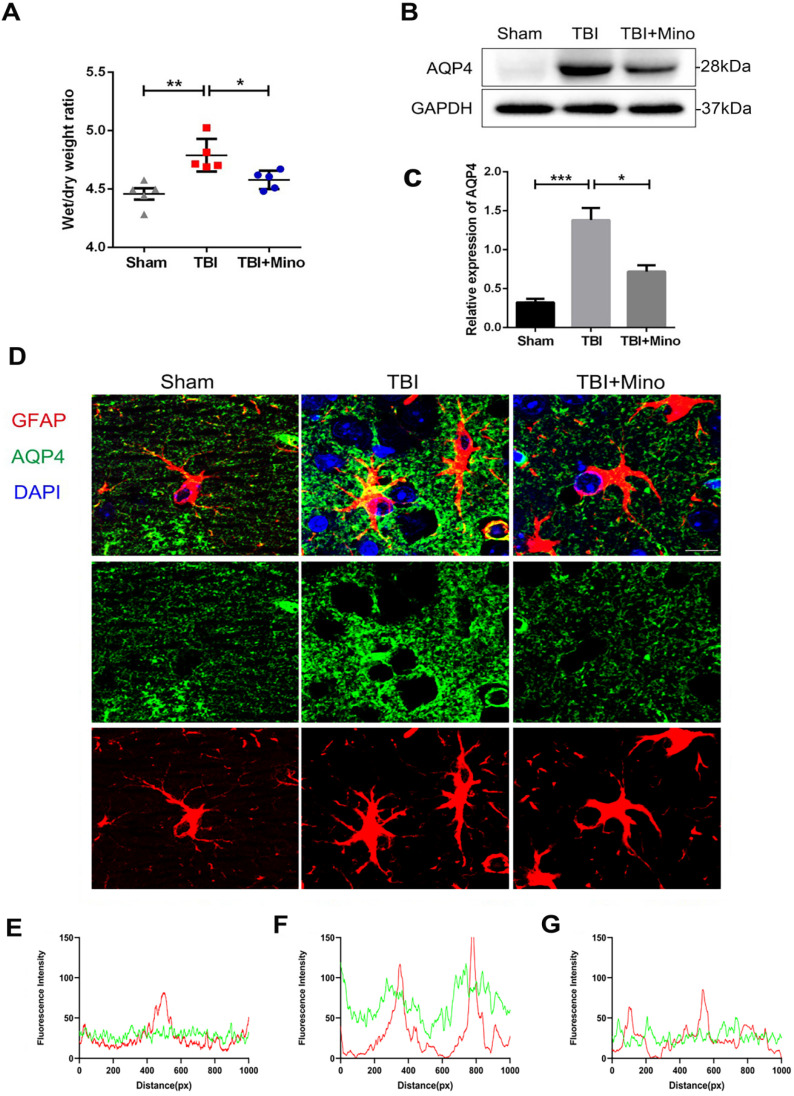
** Minocycline inhibits AQP4 expression and lessens the cerebral edema after TBI. (A)** Wet/dry weight ratios of Sham, TBI and TBI+Mino mice in the ipsilateral cortex at day 3 after TBI. n=5 per group. *P < 0.05, **P < 0.01 *vs.* the indicated group. **(B-C)** Western blot analysis and quantification of AQP4 in ipsilateral cortex 3 day after TBI. n=3 per group. *P < 0.05, ***P < 0.001 *vs.* the indicated group. **(D)** Representative immunofluorescence images depicting GFAP (red) with AQP4 (green) at 3 dpi. Scale bar = 10 µm, n=3 per group. Six images below are used to describe the specific locations of AQP4 on astrocytes. **(E-G)** The overlap of fluorescence intensity peaks along profiles to show the co-localization of GFAP and AQP4 in each group. E: Sham group. F: TBI group. G: TBI+Mino group.

In current study, we have identified the protective effect of minocycline on BBB integrity after TBI. As AQP4 is closely related to brain water homeostasis and edema development, thus we firstly analyzed the role of AQP4 on cerebral edema after TBI. As shown in Figure 3A, the brain water content was significantly increased in TBI group when compared with that in Sham group, and minocycline treatment markedly reduced the ratio of wet/dry weight as well as decreasing the water content in TBI mice. These data indicated that minocycline efficiently ameliorates the cerebral edema following TBI. Next, we examined the expression of AQP4 protein in cortex from each group. Western blot analysis showed that while TBI induces a significantly increased expression of AQP4 around the perilesional cortex, minocycline markedly inhibits this up-regulation (Figure 3B, C). To explore the relationship between AQP4 and astrocytes after TBI, we performed co-immunofluorescent staining of AQP4 and GFAP (a specific astrocyte marker). As shown in Figure 3D, AQP4 displayed a distribution with punctate reactivity around astrocytic end feet in a relative normal condition. Following TBI, astrocytes around the lesion exhibited swollen cell bodies, and AQP4 were translocated from astrocytic processes to cell bodies. Noticeably, minocycline treatment reversed the above translocation of AQP4 in astrocyte foot processes. Moreover, as indicated by the overlap profiles of fluorescence intensity peaks (Figure 3E, F), significantly increased intensity of AQP4 and reduced co-localization of GFAP and AQP4 were present in TBI group compared with those in Sham group, showing a loss of AQP4 in astrocytic end feet following TBI. As expected, minocycline treatment downregulated the fluorescent intensity of AQP4 and restored the co-localization of GFAP and AQP4 (Figure 3G). Taken together, these data suggested that minocycline administration suppresses the expression of AQP4 in astrocyte foot processes following TBI.

### Minocycline administration restores astrocyte properties following TBI

**Figure 4:**
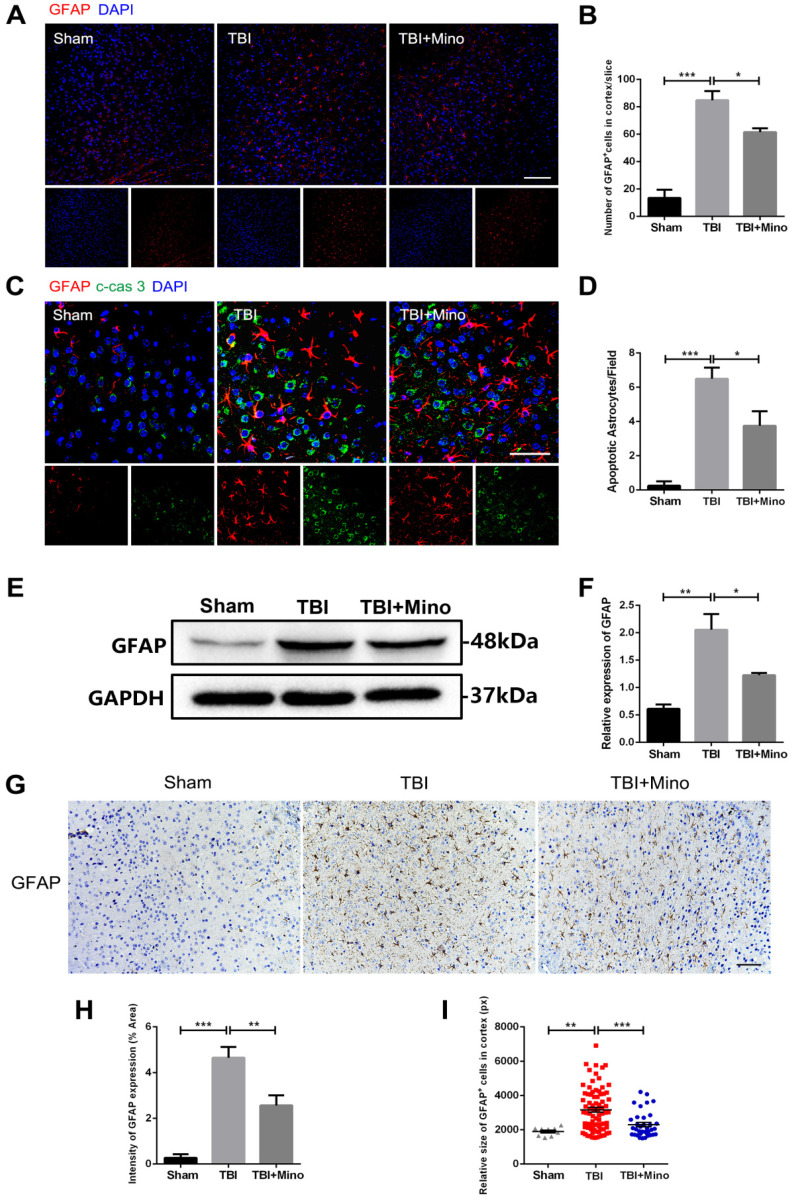
** Minocycline ameliorates astrocytosis and astrocyte swelling-induced cytotoxicity *in vivo*. (A)** Representative immunofluorescence images of GFAP (red) in perilesional cortex 3 day after TBI. Scale bar = 100 µm.** (B)** Quantification of GFAP^+^ cells in the cortex for each slice. n=4. **(C)** Representative immunofluorescence images depicting GFAP (red) with cleaved-caspase 3 (green) in perilesional cortex 3 day after TBI. Scale bar = 50 µm. **(D)** Quantification of cleaved-caspase-positive astrocytes to show the apoptotic astrocytes in the field. n=4. **(E-F)** Representative western blot analysis and quantification of GFAP in perilesional cortex 3 day post-TBI. n=3 per group. **(G)** Immunohistochemistry staining of GFAP in the cortex. Scale bar = 200 µm. **(H)** Quantification of relative intensity of GFAP positive area (% of Area). n=5 per group. **(I)** Quantification of relative size for each GFAP^+^ cells in the cortex. All data represent the mean ± SEM. *P < 0.05, **P < 0.01, ***P < 0.001 *vs.* the indicated group.

AQP4 is mainly localized in astrocytic end feet throughout the brain, and serves as a key regulator of astrocyte function 35. As described previously in current study, minocycline treatment following TBI remarkably inhibited the expression of AQP4, suggesting an underlying role of minocycline in restoring astrocyte properties. Here, we further determined the possible effect of minocycline on astrocytes. As depicted in Figure 4A and 4B, TBI caused the increased expression of GFAP in perilesional cortex, and minocycline treatment following TBI partly restored the GFAP expression. Likewise, the western blot analysis revealed that minocycline inhibits TBI-caused astrocyte activation, as indicated by the GFAP expression pattern (Figure 4E, F). Furthermore, immunohistochemistry staining of GFAP was performed to analyze the morphology and quantity of astrocytes in each group (Figure 4G). As illustrated in Figure 4H, these data based on the GFAP expression were consistent with the results described above. In addition to the changes in astrocyte quantity, we found that TBI caused an increase in the size of astrocytes, suggesting that the astrocytes were swelling. Clearly, minocycline administration partly alleviated the each increased volume of astrocytes to avoid over-swelling induced dysfunction and cytotoxicity (Figure 4I). In addition, we examined the astrocyte apoptosis *in vivo* by detecting immunofluorescence co-localization of GFAP and cleaved-caspase 3. In the experiments, quantification was performed on cleaved-caspase 3 positive astrocytes in four independent fields for each of the three groups. As evident in Figure 4C and 4D, TBI induced astrocyte apoptosis, whereas minocycline treatment following TBI significantly inhibited it. All together, these results indicated that minocycline reduces astrocytosis and astrocyte swelling following TBI to maintain normal function of astrocytes.

### Minocycline preserves BBB integrity through inhibiting the AQP4 expression

**Figure 5:**
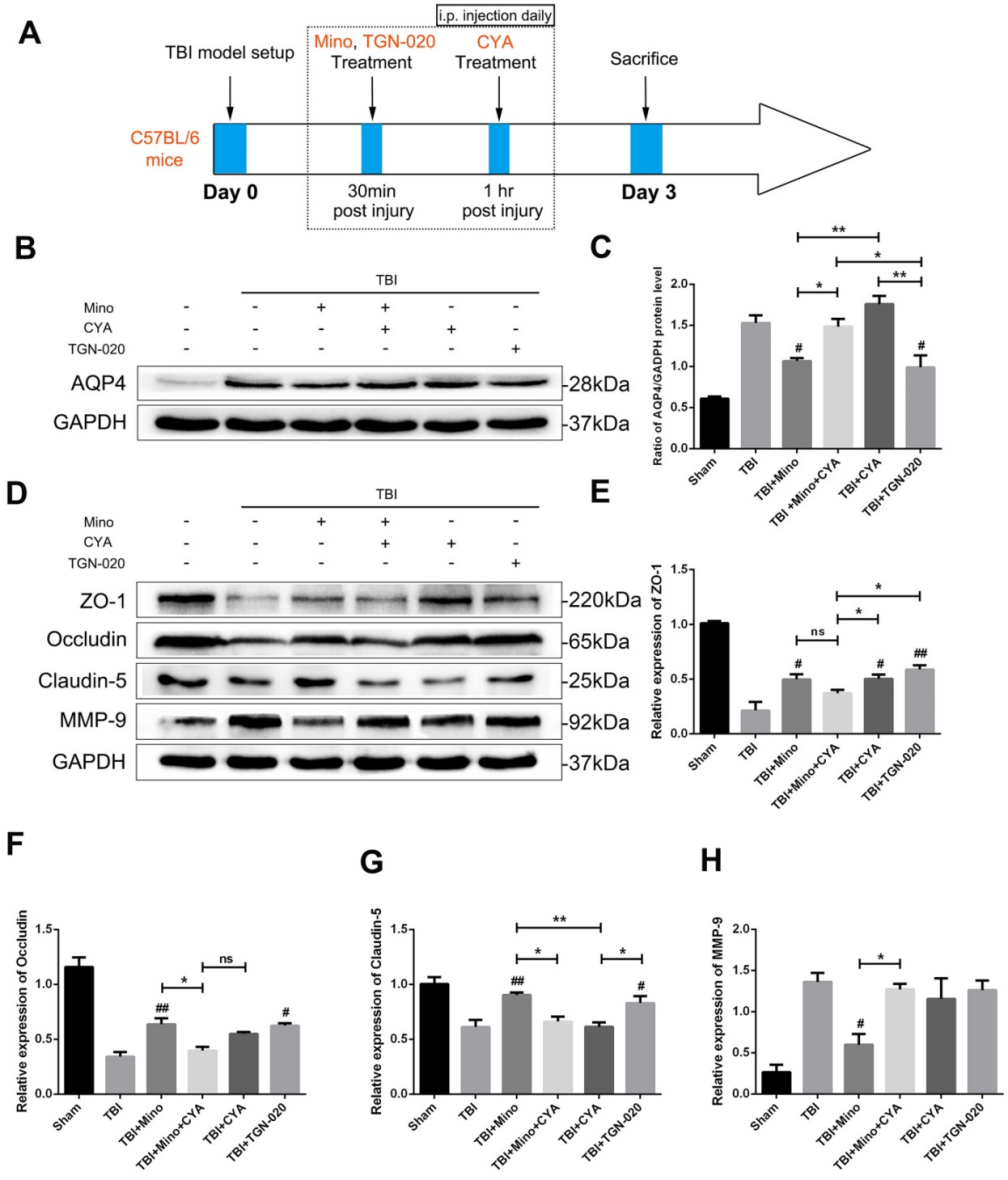
** Minocycline preserves BBB integrity through inhibiting the AQP4 expression. (A)** Abridged general view of drug administrations and experiment protocol to further explore the specific role of AQP4 after TBI. **(B-C)** Representative western blot analysis and quantification of AQP4 to verify the effects of AQP4 agonist and inhibitor after TBI. n=3 per group. **(D)** Representative western blot analysis of tight junction proteins (ZO-1, Occludin and Claudin-5) and MMP-9 in six independent groups described above. **(E-H)** Quantification of tight junction proteins and MMP-9. n=3 per group. All data represent the mean ± SEM. *P < 0.05, **P < 0.01 *vs.* the indicated group. ^#^ P < 0.05, ^##^ P < 0.01 *vs.* the TBI group.

We further investigated the specific role of AQP4 in maintaining BBB integrity related to TBI by using AQP4 agonist (cyanamide, CYA) and AQP4 inhibitor (TGN-020), which have been reported to regulate AQP4 expression 36, 37. The time points of drug administration were shown in Figure 5A. Firstly, we verified the effects of three drugs on AQP4 expression by western blot analysis. As depicted in Figure 5B and 5C, the expression of AQP4 was significantly upregulated in the co-treatment group of minocycline and cyanamide relative to the minocycline group. Compared with the minocycline group, the cyanamide group displayed a significantly increased expression of AQP4, whereas there was no significant difference in the expression of AQP4 between the TBI+CYA group and the TBI group (Figure 5B and C). On the contrary, TGN-020 treatment following TBI markedly downregulated the expression of AQP4 (Figure 5B and C). Based on these positive results, we next explored the BBB integrity by examining the expression of tight junction proteins and MMP-9 (Figure 5D). As shown in Figure 5E-G, either minocycline or TGN-020 was able to significantly upregulate the tight junction proteins (ZO-1, Occludin and Claudin-5). The co-treatment group of minocycline and cyanamide displayed a reduced expression of tight junction proteins relative to the minocycline group. Interestingly, compared with the TBI group, cyanamide treatment significantly enhanced the expression of ZO-1 and led to a slight increase in Occludin, which showed no statistical significance. These results suggested a BBB protective role of minocycline in our model. Meanwhile, neither cyanamide nor TGN-020 exhibited a regulatory activity towards MMP-9 expression level *in vivo* (Figure 5H). In summary, these data led us to propose that minocycline preserves BBB integrity through inhibiting AQP4 level *in vivo*.

### Minocycline mediates astrocyte properties via regulation of AQP4

**Figure 6:**
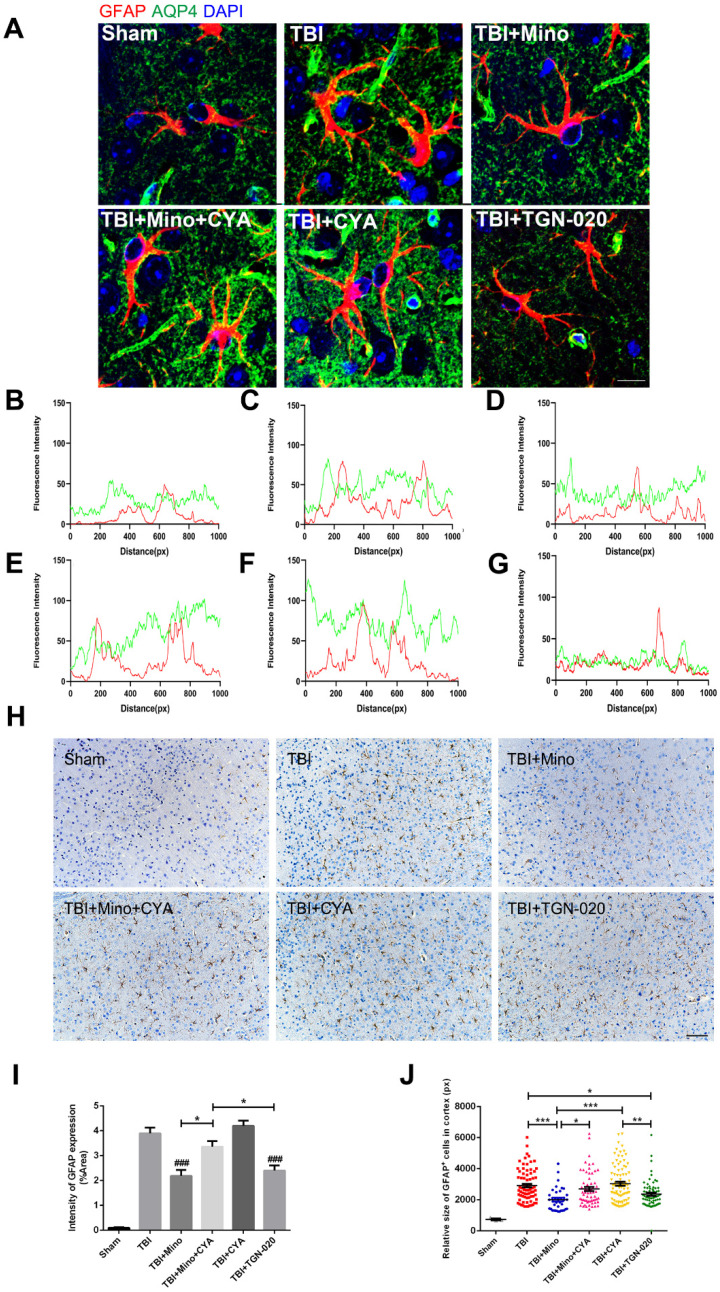
** Minocycline mediates astrocytic properties through regulation of AQP4 level *in vivo*. (A)** Representative immunofluorescence images depicting GFAP (red) with AQP4 (green) at 3 dpi in six independent groups. Scale bar = 10 µm. **(B-G)** The overlap of fluorescence intensity peaks along profiles to show the co-localization of GFAP and AQP4 in each group. B: Sham group. C: TBI group. D: TBI+Mino group. E: TBI+Mino+CYA group. F: TBI+CYA group. G: TBI+TGN-020 group. **(H)** Immunohistochemistry staining of GFAP in the perilesional cortex. Scale bar = 200 µm. **(I)** Quantification of relative intensity of GFAP positive area (% of Area). n=5 per group. ^###^ P < 0.001 *vs.* the TBI group.** (J)** Quantification of relative size for each GFAP^+^ cells in the perilesional cortex. All data represent the mean ± SEM. *P < 0.05, **P < 0.01, ***P < 0.001* vs.* the indicated group.

To further investigate the specific role of AQP4 in astrocyte properties related to TBI, we performed double immunofluorescent staining of GFAP (red) and AQP4 (green) as well as immunohistochemistry of GFAP in the six groups described above. As depicted in Figure 6A-G, combined treatment of minocycline and cyanamide following TBI caused a translocation of AQP4 to the astrocyte cell bodies compared with the minocycline group; this translocation was predominantly observed in the cyanamide group after TBI. TGN-020 treatment following TBI led to a distribution of AQP4 around astrocytic processes, which was similar with the observation in minocycline group. In the meantime, compared with the TBI+Mino group, the TBI+Mino+CYA group exhibited a compromised co-localization of AQP4 and GFAP, as evident in the overlap profiles of fluorescence intensity peaks (Figure 6D, E). Notably, cyanamide treatment alone following TBI displayed a better co-localization of these two proteins compared with TBI+Mino+CYA group, while there was no significant difference in the co-localization of them between the CYA group and the TBI group (Figure 6C, E, F). As shown in Figure 6D and G, treatment with either minocycline or TGN-020 following TBI restored the delocalization of AQP4 and GFAP. Furthermore, the immunohistochemistry of GFAP was performed to assess the morphology and quantity of astrocytes in each of the six groups (Figure 6H). As illustrated in Figure 6I, both minocycline and TGN-020 significantly downregulated the relative intensity of GFAP expression compared with the TBI group, and a stronger intensity was found in co-treatment group of minocycline and cyanamide relative to the minocycline group. Moreover, we observed that minocycline and TGN-020 treatment following TBI significantly reduced the relative size of each GFAP^+^ cells, which implicated a preferable effect in regulation of astrocyte swelling (Figure 6J). Besides, combined treatments with cyanamide and minocycline markedly enlarged the astrocyte size relative to the minocycline group (Figure 6J). All these data implied that minocycline reduces astrocyte swelling and optimized AQP4 localization through regulating the AQP4 level *in vivo*.

### Minocycline mitigates glutamate-induced primary cultured astrocyte swelling *in vitro*

**Figure 7:**
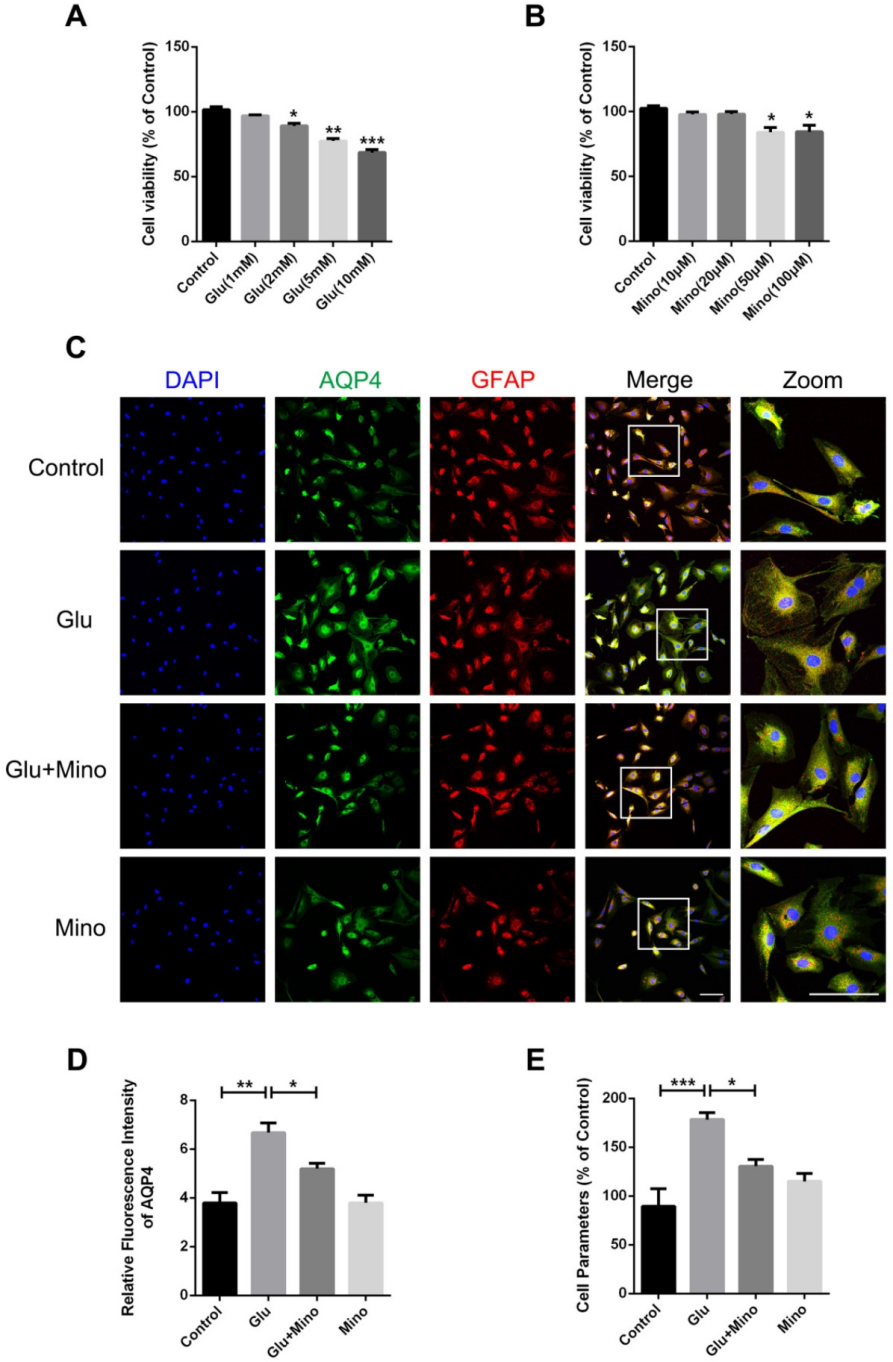
** Minocycline mitigates glutamate-induced astrocyte swelling *in vitro*. (A)** Cell viability assay of different concentrations of glutamate treatment (1, 2, 5, 10 mM) for 48 h to test cytotoxicity of glutamate on primary cultured astrocytes. n=6 per group. *P < 0.05, **P < 0.01, ***P < 0.001 *vs.* the control group.** (B)** Cell viability assay of different concentrations of minocycline treatment (10, 20, 50, 100 µM) for 2 h. n=6 per group. *P < 0.05 *vs.* the control group.** (C)** Representative images of immunofluorescence staining depicting GFAP (red) with AQP4 (green) in astrocytes after glutamate and minocycline treatment. Scale bar= 100 µm.** (D)** Relative fluorescence intensity of AQP4 in four independent groups described above. n =3 per group. *P < 0.05, **P < 0.01 *vs.* the indicated group. **(E)** Quantitative analysis of image data from **(C)** to represent the astrocyte volume by cell parameter method. n=6 per group. *P < 0.05, ***P < 0.001 *vs.* the indicated group. All data represent the mean ± SEM.

Here, we further investigated the effect of minocycline treatment on astrocyte swelling induced by glutamate *in vitro* 38. Firstly, we determined the cytotoxicity of different concentrations of glutamate and minocycline by using the CCK-8 assay. As depicted in Figure 7A, treatment of primary cultured astrocytes with 2, 5 or 10 mM glutamate led to a significant decrease of cell viability compared with the control group, whereas 1 mM glutamate had no effect on cell viability. In the meantime, while 50 and 100 μM minocycline significantly reduced the cell viability, 10 μM and 20 μM showed no cytotoxicity relative to the control group (Figure 7B). Based on these results, we chose glutamate (1 mM) and minocycline (20 μM) for further analysis. Immunofluorescence staining of GFAP and AQP4 was performed on primary cultured astrocytes treated with minocycline and glutamate (Figure 7C). As indicated by quantification immunofluorescence intensity of AQP4, glutamate significantly enhanced the intensity of AQP4, while pretreatment of the cells with minocycline blocked this enhancement (Figure 7D). Given that a perimeter method has been shown to assess the cell volume accurately 39, we employed this perimeter method for evaluating astrocyte volume in each group. As shown in Figure 7E, glutamate significantly enlarged astrocyte volume compared with the control group, whereas minocycline pretreatment efficiently ameliorated glutamate-induced astrocyte swelling. These results demonstrated that minocycline mitigates glutamate-induced astrocyte swelling *in vitro*.

### AQP4 siRNA abolishes glutamate-induced astrocyte swelling *in vitro*

**Figure 8:**
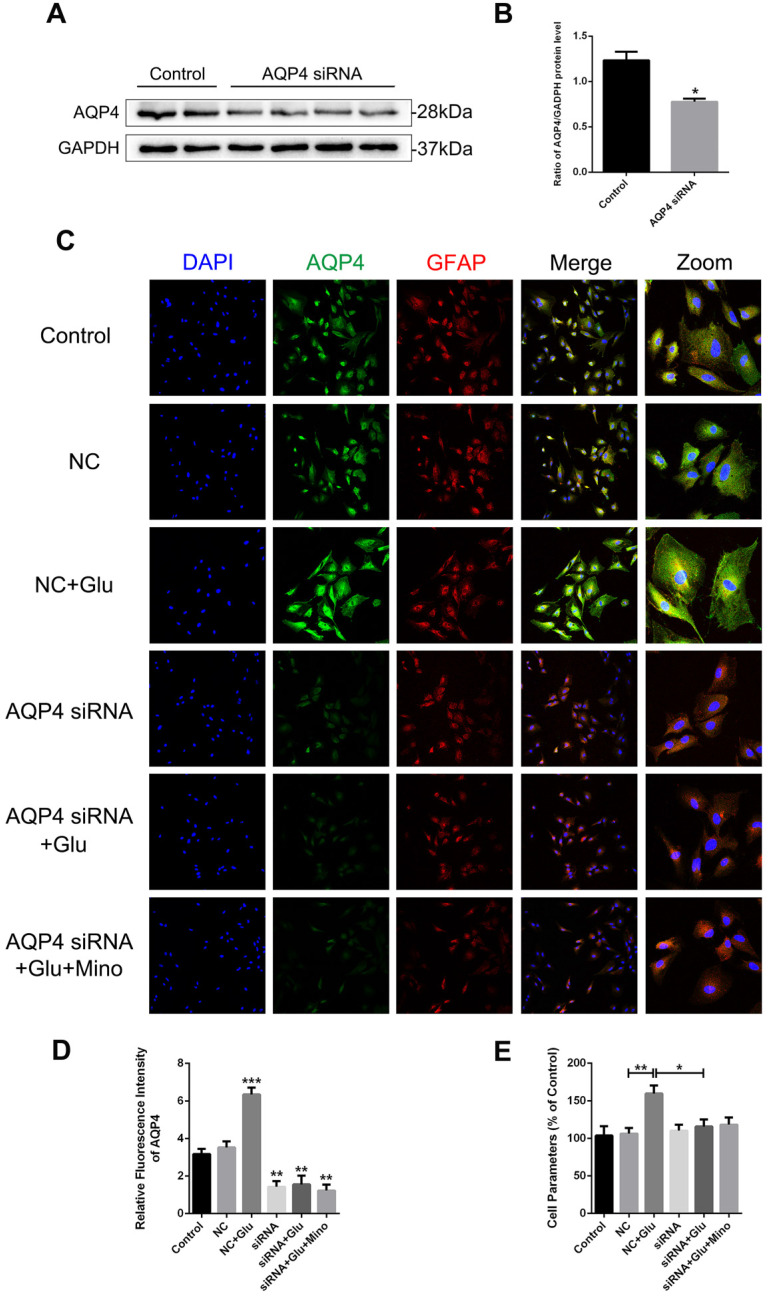
** AQP4 siRNA abolishes glutamate-induced astrocyte swelling *in vitro*. (A, B)** Western blot analysis and quantification of AQP4 expression between the control group and AQP4 siRNA treated group. n=4 per group. *P < 0.05 *vs.* the control group. **(C)** Representative images of immunofluorescence staining depicting GFAP (red) with AQP4 (green) in primary culture astrocytes after incubation with AQP4 siRNA, glutamate and minocycline, respectively. NC represented negative control. Scale bar=100 µm. **(D)** Quantification of fluorescence intensity of AQP4 in each group. n=3 per group. *P < 0.05, ***P < 0.001 *vs.* the control group. **(E)** Quantitative analysis of image data from **(C)** to represent the astrocyte volume by cell parameter method. n=6 per group. *P < 0.05, **P < 0.01 *vs.* the indicated group. All data represent the mean ± SEM.

To determine whether AQP4 is involved in the regulation of minocycline for ameliorating astrocyte volume, we utilized the AQP4 siRNA transfection to knockdown AQP4 level in primary cultured astrocytes. As evident in the western blot analysis and quantification assay, AQP4 siRNA significantly reduced the AQP4 expression in astrocytes (P = 0.0461 < 0.05, Figure 8A and B). Similar results were observed in quantification of fluorescence intensity of AQP4 in each group (Figure 8D). Collectively, these data indicated that the siRNA transfection used in this study effectively knocked down the expression of AQP4 in the cells. We then analyzed whether reduced expression of AQP4 affects the swelling of astrocytes treated with minocycline or glutamate *in vitro*. As shown in Figure 8C, the negative control (NC) group showed no differences with the control group in the size of astrocytes, albeit glutamate-induced astrocyte swelling was evident in both groups. However, no enlargement in the size of astrocytes was found in the AQP4 knockdown group, indicating that knockdown of AQP4 abolished glutamate-induced astrocyte swelling (Figure 8C). Besides, minocycline showed no effect on the astrocytes expressing AQP4 siRNA (Figure 8D). Consistently, similar results in astrocyte volume were obtained based on the perimeter method (Figure 8E). All these data indicated that astrocyte swelling is mediated by AQP4 expression, and AQP4 knockdown abolished the effect of minocycline in the control of the astrocyte volume.

## Discussion

Recently, there have been numerous findings in BBB repair in the pathophysiological process of CNS disorders, however, a comprehensive understanding of BBB and efficacious therapeutic strategies for brain injuries are still lacking. In this study, we mainly focused on the neuroprotective effect of minocycline on BBB function and cerebral edema following TBI. We have shown that minocycline, a potent antibiotic used in the clinic, attenuates BBB disruption and astrocytic edema in the TBI mice through suppressing AQP4 level. Meanwhile, minocycline was found to restore AQP4 localization in perivascular astrocytic endfeet, which was correlated with the gliovascular interactions in BBB properties.

Numerous studies have shown that minocycline treatment has the characteristic of protecting the integrity of BBB. Previous studies found that minocycline treatment significantly reduces BBB permeability by enhancing the expressions of tight junction proteins, and promoted neurovascular remodeling after cerebral ischemic injury 24. Also, minocycline was shown to protect BBB integrity through inhibiting tight junction loss as well as MMP-9 up-regulation in human brain microvascular endothelial cells under hypoxia 40. In intracerebral hemorrhage model rats, minocycline administration effectively reversed IgG extravasation and edema induced by BBB breakdown 41. In the present study, we observed that minocycline reduces the leakage of sodium fluorescein and Albumin in the ipsilateral cortex 3 days after TBI. Moreover, minocycline treatment following TBI markedly restored the loss of tight junction proteins (ZO-1, Occludin and Claudin-5) and endothelial coverage of ZO-1 in mice (Figure 2). Thus, this study further provided evidence that minocycline administration is beneficial to alleviate TBI-induced BBB dysfunction in mice.

As a representative pathological process after TBI, brain edema is closely interacted with BBB integrity in the progression of secondary injuries 42. Minocycline has been reported to attenuate brain edema in various CNS injuries, including intracerebral hemorrhage 43 and TBI 44. Consistent with the previous reports, we found that minocycline remarkedly ameliorates TBI-induced brain edema (assessed by water content). Cerebral edema has been categorized into cytotoxic edema (cellular edema) and vasogenic edema, each of which occurs at different time points post injury 45. Recent studies revealed that TBI encompasses those two types of edema in the pathological development, causing contradiction in edema intervention 42. It is known that AQP4, water channel protein, is responsible for mediating both types of edema that concomitantly contribute to TBI progression 46. It has been reported that inhibition of AQP4 relieves water transport to intracellular space and alleviates the formation of cytotoxic edema 37. All kinds of cells in the CNS are likely to induce cytotoxic edema following various injuries, especially in astrocytes 47. Evidence demonstrated that pretreated with AQP4 inhibitor significantly blocks water content elevation and tissue edema after spinal cord injury 48. AQP4 down-regulation by depletion of relevant transcription factors attenuates cytotoxic edema in perilesional cortex 24h after TBI 49. Consistent with the previous research 50, we found that minocycline treatment reduces the expression of AQP4 in perilesional cortex at 3 dpi (Figure 3B, C). Furthermore, we examined the astrocyte properties at the same time point and observed that minocycline mitigates astrocyte swelling as well as over swelling-induced cytotoxicity (Figure 4). All these results implied that minocycline effectively ameliorates cytotoxic edema through down-regulating AQP4 expression *in vivo*.

In addition to the *in vivo* assays, primary cultured astrocytes were selected to simulate cytotoxic edema *in vitro*, which were characterized by astrocyte swelling and cell volume enlargement. Glutamate-induced astrocyte swelling has been proved to be under the control of AQP4 expression and AQP4 deletion deprived the volume alteration in primary rat astrocytes 38. A widely used AQP4 inhibitor acetazolamide was found to mitigate cellular swelling and edema in astrocytic stretching injury model following mTBI 51. In the present study, pretreatment with minocycline effectively reduced the enlargement of astrocyte volume (Figure 7). Meanwhile, glutamate-induced astrocyte swelling induced by glutamate was abolished by AQP4 knockdown and the regulatory effect of minocycline on astrocyte volume *in vitro* was undetected in this circumstance (Figure 8). In contrast to alleviating cytotoxic edema, inhibition of AQP4 expression was found to exacerbate the formation of vasogenic edema and BBB breakdown 45. It appears that AQP4 localization at astrocytic end feet plays an important role in vasogenic edema clearance and BBB integrity 52, 53. Following TBI, AQP4 was translocated from its original localization around perivascular astrocytic processes to astrocytic soma; this translocation may contribute more to pathogenesis of vasogenic edema compared with AQP4 expression level in perilesional cortex 54. Similar translocation of AQP4 was observed in post-stroke white matter injury, implying that AQP4 translocation acts an indicator gliovascular dysfunction and BBB disruption 15, 55. Acetazolamide, a canonical AQP4 inhibitor, was reported to prevent the depolarization of astrocytic AQP4 following TBI in mice 56, 57. We therefore examined the localization of AQP4 and found that while TBI caused translocation of AQP4 from astrocytic end processes to astrocytic cell bodies, minocycline treatment markedly reversed this translocation (Figure 3). Besides, we noticed that minocycline inhibited TBI-induced astrocyte activation after TBI (Figure 4A, E). Given that microglia is capable to alter astrocyte response in brain injury 32, we proposed that an indirect inhibitory effect of minocycline on microglia may underlie the above observation. Thus, minocycline mediated cerebral edema and BBB disruption via the two distinct mechanisms: inhibitory effect on AQP4 and optimizing function of AQP4 localization.

As a crucial modulator in water homeostasis, AQP4 is involved in the neuroprotection in various kind of CNS disorders 9, 58. AQP4 is specifically enriched around astrocyte processes, making it indispensable in neurogliovascular systematic interactions, especially in BBB structure and gliovascular coupling 37, 59. Tang et al has reported that suppression of AQP4 improves the maintenance of BBB integrity as well as neurological outcomes after cerebral ischemia 14. Likewise, down-regulation of perivascular AQP4 after TBI effectively decreased BBB vascular permeability in mice 60. In this study, we used AQP4 agonist cyanamide 36 and specific AQP4 inhibitor TGN-020 61 to modulate AQP4 level in the TBI mice. Our current study revealed that TGN-020 and minocycline displays similar capacity in up-regulating tight junction proteins following TBI, indicating that the inhibitory effect of minocycline on AQP4 is involved in regulating BBB function. Meanwhile, we observed that cyanamide aggravates tight junction loss due to minocycline treatment. Interestingly, cyanamide treatment alone following TBI significantly enhanced the expression of ZO-1 and caused a slight increase in Occludin (Figure 5). Thus, upregulation of AQP4 may restore BBB function as well. More evidence supported our hypothesis that Apelin-13 exerts a BBB protective role for cerebral ischemic diseases through promoting AQP4 expression *in vivo* 62. Meanwhile, sulforaphane administration up-regulated AQP4 levels, leading to more water clearance against edema formation and BBB dysfunction following TBI 63. Hence, the relationship between more specific expression level of AQP4 and the corresponding effect of BBB integrity needs to be further investigated. Besides, AQP4 localization and astrocyte swelling were detected in the six groups indicated in Figure 6. The results showed that TGN-020 treatment blocks the increase in astrocyte volume. By contrast, treatment with cyanamide aggravated astrocyte swelling and displayed no change in AQP4 localization, which is consistent with the previous reports on the effect of minocycline treatment in cytotoxic and vasogenic edema. Thus, we concluded that minocycline regulates BBB and astrocyte properties through regulating AQP4 expression and localization after TBI in mice.

Overall, our study illustrated that minocycline inhibits TBI-induced elevated AQP4 level, and protects BBB integrity and regulates astrocyte properties following TBI. Our current study led us to identify AQP4 as a new target for minocycline treatment in TBI, thereby providing an alternative therapeutic strategy for functional recovery following TBI.
